# Charge of Prof. Pancoast

**Published:** 1855-05

**Authors:** 


### CHARGE TO THE GRADUATING CLASS OF JEFFERSON MEDICAL COLLEGE.

### PROF. PANCOAST.

There is not, perhaps, in the whole routine of his duties, a more difficult task in its performance by the medical teacher, than that of framing a valedictory to the graduates. There is so much to say, and he would say so much, that the space for its performance discourages him from the effort because of its narrow limits. The history of his own trials, vicissitudes, and labors, warns him of how much of disappointment and discouragement they will be compelled to meet, and how much moral fortitude they must exercise, to be able to triumph over them; how much labor they must bestow; how much of effort they will be called upon to exercise; how many midnight lamps must be consumed in close and deep research, in order to place them upon even a *respectable footing* in the profession, to say nothing of conspicuity or prominence. All these things, and *volumes* more he would tell them, but time forbids; and the task would prove, if full justice be done to the subject, a most laborious one. The charge of Prof. PANCOAST is not only an able, but a feeling one, and is couched in the most chaste language. We had the pleasure of hearing his opening lecture last fall, and are not disappointed to find that the class has had so rich a treat in this valedictory.

We select the following, as not only appropriate, but many of the *alumni* of this institution will find in it views which have been often heretofore presented for their consideration and action:

"We are all too much given in this world to attribute the success, even of men of unquestioned genius, to that endowment. As if genius alone, great and glorious gift as to some of its possessors it has undoubtedly proven, were all in all, capable of supplying all deficiencies, even imperfect education and slothful habits of mind. This truth you may consider at least certain, that the greatest geniuses the world has ever seen, are, and so far as is known, always have been, the most industrious of men; and no one has ever been permitted to look at them behind the curtain, without finding them busy—busy—beyond the measure of all ordinary human toil.

Some of the men of greatest renown in the records of history have, from motives of policy, been inclined to attribute their success to the favoring influences of their fortune, their destiny, or their star.

But a clearer investigation into the nature of their pursuits, in the dawning period of their greatness, makes it very manifest that their fortune and their destiny were of their own carving, and their star, of which it sounded well for purposes of policy to speak, was but the student's lamp, fed to meteor brightness by vigilence and unwearying application. And all the ability, the tact, the knowledge, the ready preparedness, which looked like intuition, and was often regarded as inspiration, were but the natural fruits of a system of training and self-cultivation, which it was, I believe, the part of true genius to plan, and still greater genius to persecute so thoroughly.

Your ears may weary now with the repetition, and your hearts faint at the prospect of the unceasing activity and labor that I enjoin upon you. But bear in mind, that you will find this to be in reality no infliction. And if you were looking merely to your own happiness and enjoyment, rather than to the influence that you may be enabled to exert for the good of others, you should be induced to follow it. The range of study before you is large—and herein lies its greatest attraction that you can never exhaust it."

